# Supplementary material for: Gamma rhythms and visual information in mouse V1 specifically modulated by somatostatin+ neurons in reticular thalamus
Source: eLife. 2021 Apr 12;10:e61437. doi: 10.7554/eLife.61437 (PMC8064751; doi:10.7554/eLife.61437)
Supplement: Figure 2—source data 1. [file elife-61437-fig2-data1.docx]

|  | | **Still & laser off** | **Still & laser on** | **Running & laser off** | | **Running & laser on** | **p-value** |
| --- | --- | --- | --- | --- | --- | --- | --- |
| **Fig. 2-S3C (n=43 channels)** | θ | 10.4 | 11.9 | |  |  | 1.1e-3 |
|  | β | 14.6 | 19.4 | |  |  | 9.2e-8 |
|  | γ | 21.8 | 24.1 | |  |  | 1.0e-4 |
| **Fig. 2-S3D (n=43 channels)** | θ | 10.4 |  | | 7.4 |  | 1.4e-8 |
|  | β | 14.6 |  | | 9.7 |  | 5.8e-8 |
|  | γ | 21.8 |  | | 29.2 |  | 1.0e-7 |
| **Fig. 2-S3E (n=189 channels, 4 mice)** | θ | -0.2 | -0.7 | | -0.4 | -0.4 | 0.53 (still), 0.26 (running) |
|  | β | 0.7 | 1.5 | | -1.0 | 2.2 | 0.26 (still), 0.15 (running) |
|  | γ | 0.8 | 0.9 | | -2.5 | 0.5 | 0.63 (still), 0.21 (running) |
| **Fig. 2-S3H (n=99 channels)** | θ | 0.5 | -6.2 | |  |  | 4.4e-10 |
|  | β | 7.6 | 5.7 | |  |  | 5.2e-2 |
|  | γ | 3.6 | -2.9 | |  |  | 1.1e-25 |
| **Fig. 2-S3I (n=154 channels, 4 mice)** | θ | -0.18 | -0.72 | | -0.58 | -0.59 | 0.41 (still), 0.41 (running) |
|  | β | 0.45 | 1.8 | | -1.3 | 2.2 | 0.52 (still), 0.03 (running) |
|  | γ | 0.51 | 0.54 | | -2.6 | 0.52 | 0.47 (still), 0.05 (running) |
| **Fig. 2-S3K (n=191 BS, 113 NS, in 4 mice)** | BS | 9.1 ± 1.22 | 11.9 ± 1.31 | | 15.0 ± 0.77 | 18.2 ± 1.00 | 0.10 (still), 0.002 (running) |
|  | NS | 11.4 ± 1.44 | 15.2 ± 0.96 | | 22.2 ± 2.45 | 27.8 ± 3.07 | 0.011 (still), 0.010 (running) |
| **Fig. 2-S3L (n=100 BS, 32 NS cells, in 4 mice)** | BS | 3.34 ± 0.22 | 8.16 ± 1.88 | | 12.3 ± 5.04 | 24.6 ± 8.52 |  |
|  | NS | 8.5 ± 1.52 | 15.4 ± 3.67 | | 10.4 ± 4.35 | 17.15 ± 7.10 |  |
| **Fig. 5-S1C (n=102 channels)** | θ | 54 | 49 | |  |  | 9.2e-3 |
|  | β | 63 | 77 | |  |  | 5.2e-5 |
|  | γ | 90 | 146 | |  |  | 8e-18 |
| **Fig. 5-S1D (n=102 channels)** | θ | 54 |  | | 49 |  | 0.01 |
|  | β | 63 |  | | 68 |  | 0.48 |
|  | γ | 90 |  | | 197 |  | 3.8e-24 |
| **Fig. 5-S1E (n=313 channels, 4 mice)** | θ | 45 | 44 | | 56 | 43 | 0.43 (still), 0.34 (running) |
|  | β | 66 | 70 | | 65 | 67 | 0.62 (still), 0.91 (running) |
|  | γ | 97 | 111 | | 130 | 143 | 0.11 (still), 0.19 (running) |
| **Fig. 5-S1H (n=59 channels)** | θ | 6 | -21 | |  |  | 3e-13 |
|  | β | 20 | 7 | |  |  | 2.4e-10 |
|  | γ | 16 | 74 | |  |  | 5e-10 |
| **Fig. 5-S1I (n=267 channels, 4 mice)** | θ | 8.7 | -0.9 | | 8.2 | -2.0 | 0.32 (still), 0.06 (running) |
|  | β | 22 | 18 | | 13 | 4 | 0.27 (still), 0.67 (running) |
|  | γ | 4.0 | 32 | | 12 | 25 | 0.02 (still), 0.21 (running) |
| **Fig. 5-S1K (n=58 BS, 44 NS, in 4 mice)** | BS | 8.0 | 9.8 | | 11.3 | 13.4 | 0.01 (still), 4e-4 (running) |
|  | NS | 20.4 | 29.5 | | 31.6 | 41.9 | 2e-3 (still), 8e-5 (running) |
| **Fig. 5-S1L (n=93 BS, 22 NS cells, in 4 mice)** | BS | 11.45 ± 5.82 | 37.62 ± 8.18 | | 16.6 ± 2.41 | 30.2 ± 3.77 |  |
|  | NS | 7.3 ± 2.26 | 17.2 ± 4.11 | | 11.1 ± 3.50 | 22.48 ± 6.03 |  |
| **Fig. 5-S2C (n=34 channels)** | θ | 9.8 | 9.8 | |  |  | 0.51 |
|  | β | 16.1 | 18.2 | |  |  | 3.6e-7 |
|  | γ | 24.6 | 30.5 | |  |  | 2.2e-8 |
| **Fig. 5-S2D (n=34 channels)** | θ | 9.8 |  | | 10.4 |  | 0.07 |
|  | β | 16.1 |  | | 17.9 |  | 4.0e-7 |
|  | γ | 24.6 |  | | 47.1 |  | 8.1e-8 |
| **Fig. 5-S2E (n=121 channels, 2 mice)** | θ | 12 | 12 | | 13 | 20 | 0.98 (still), 0.72 (running) |
|  | β | 16 | 17 | | 17 | 17 | 0.81 (still), 0.61 (running) |
|  | γ | 25 | 29 | | 53 | 55 | 0.76 (still), 0.49 (running) |
| **Fig. 5-S2H (n=37 channels)** | θ | 0.3 | 0.3 | |  |  | 0.03 |
|  | β | 0.2 | 0.4 | |  |  | 4.5e-9 |
|  | γ | 0.3 | 0.7 | |  |  | 2.4e-9 |
| **Fig. 5-S2I (n=267 channels, 2 mice)** | θ | 0.16 | 0.17 | | -0.21 | 0.09 | 0.65 (still), 0.58 (running) |
|  | β | 0.02 | 0.56 | | -0.19 | 2.13 | 0.11 (still), 0.43 (running) |
|  | γ | 0.57 | 0.0 | | -0.87 | -0.62 | 0.08 (still), 0.13 (running) |
| **Fig. 5-S2K (n=75 BS, 126 NS, in 2 mice)** | BS | 6.7 | 7.0 | | 8.8 | 9.4 | 0.22 (still), 0.07 (running) |
|  | NS | 8.1 | 9.5 | | 11.7 | 13.1 | 0.027 (still), 0.04 (running) |
| **Fig. 5-S2L (n=24 BS, 22 NS cells, in 2 mice)** | BS | 8.15 ± 0.45 | 9.9 ± 1.22 | | 8.34 ± 1.97 | 13.7 ± 2.12 |  |
|  | NS | 7.61 ± 1.18 | 10.8 ± 1.09 | | 7.73 ± 2.12 | 9.91 ± 2.71 |  |

**Figure 2—source data 1.** Results of significance testing across different conditions. Power amplitudes are in units of 1,000*uV^2^/Hz and firing rates are in Hz. BS: broad-spiking, NS: narrow-spiking.
