# Supplementary material for: Gamma rhythms and visual information in mouse V1 specifically modulated by somatostatin+ neurons in reticular thalamus
Source: eLife. 2021 Apr 12;10:e61437. doi: 10.7554/eLife.61437 (PMC8064751; doi:10.7554/eLife.61437)
Supplement: Figure 7—source data 1. [file elife-61437-fig7-data1.docx]

|  | | **Still & laser off** | **Still & laser on** | **Running & laser off** | | **Running & laser on** | **p-value** |
| --- | --- | --- | --- | --- | --- | --- | --- |
| **Fig. 7-S1C (n=38 channels)** | θ | 346 | 289 | |  |  | 1.1e-6 |
|  | β | 981.4 | 733.1 | |  |  | 1.6e-5 |
|  | γ | 1915.4 | 1398.3 | |  |  | 5.4e-4 |
| **Fig. 7-S1D (n=38 channels)** | θ | 346 |  | | 451.1 |  | 5.4e-10 |
|  | β | 981.4 |  | | 1072.5 |  | 2.2e-11 |
|  | γ | 1915.4 |  | | 2213.4 |  | 6.3e-23 |
| **Fig. 7-S1E (n=88 channels, 3 mice)** | θ | 415.3 | 388.5 | | 781.0 | 797.6 | 0.18 (still), 0.63 (running) |
|  | β | 644.8 | 527.9 | | 857.4 | 772.1 | 0.11 (still), 0.14 (running) |
|  | γ | 1160.4 | 991.5 | | 1227.9 | 1184.4 | 0.04 (still), 0.19 (running) |
| **Fig. 7-S1H (n=99 channels )** | θ | -45.1 | -24.9 | |  |  | 0.52 |
|  | β | -57.1 | -127.8 | |  |  | 5.5e-13 |
|  | γ | 13.6 | -243.4 | |  |  | 1.4e-16 |
| **Fig. 7-S1I (n=76 channels, 3 mice)** | θ | 2.8 | -47.3 | | 52.7 | -93.8 | 0.09 (still), 0.007 (running) |
|  | β | -22.6 | -90.4 | | 38.8 | -95.3 | 0.04 (still), 3.2e-3 (running) |
|  | γ | 5.5 | -152.1 | | 32.7 | -133.4 | 0.001 (still), 5.5e-4 (running) |
| **Fig. 7-S1K (n=158 MU, 35 SU, in 3 mice)** | MU | 13.1 | 6.2 | | 15.1 | 8.0 | 6e-7 (still), 6e-9 (running) |
|  | SU | 10.0 | 7.5 | | 14.4 | 8.4 | 0.013 (still), 7e-3 (running) |
| **Fig. 7-S1L (n=55 MU, 29 SU cells, in 3 mice)** | MU | -32 | -27 | | -28 | -53 |  |
|  | SU | -33 | -25 | | -39 | -21 |  |
| **Fig. 7-S2C (n=18 channels)** | θ | 575.2 | 609.5 | |  |  | 3.2e-4 |
|  | β | 1021.0 | 1166.2 | |  |  | 1.9e-4 |
|  | γ | 3137.6 | 3335.4 | |  |  | 1.9e-4 |
| **Fig. 7-S2D (n=18 channels)** | θ | 491.2 |  | | 878.8 |  | 6.1e-9 |
|  | β | 1495.0 |  | | 1121.6 |  | 4.9e-6 |
|  | γ | 3201.8 |  | | 3409 |  | 0.36e |
| **Fig. 7-S2E (n=92 channels, 3 mice)** | θ | 442.1 | 364.5 | | 752.4 | 760.1 | 0.08 (still), 0.82 (running) |
|  | β | 665.5 | 134.8 | | 882.3 | 748.3 | 0.94 (still), 0.70 (running) |
|  | γ | 1120.1 | 1299.1 | | 1234.8 | 1169.8 | 0.56 (still), 0.62 (running) |
| **Fig. 7-S2H (n=19 channels )** | θ | -147.5 | 39.8 | |  |  | 4.5e-6 |
|  | β | -208.1 | -31.3 | |  |  | 1.8e-11 |
|  | γ | -285.6 | 79.9 | |  |  | 6.4e-19 |
| **Fig. 7-S2I (n=81 channels, 3 mice)** | θ | -83.1 | -27.4 | | -31.1 | 49.8 | 0.04 (still), 0.04 (running) |
|  | β | -109.2 | -88.3 | | -96.7 | -28.8 | 0.44 (still), 0.10 (running) |
|  | γ | 112.1 | 127.3 | | 171.4 | 229.5 | 0.56 (still), 0.23 (running) |
| **Fig. 7-S2K (n=85 BS, 49 NS, in 3 mice)** | MU | 9.4 | 11.3 | | 11.4 | 14.2 | 4e-4 (still), 9e-5 (running) |
|  | SU | 11.1 | 12.3 | | 13.8 | 15.1 | 0.31 (still), 0.22 (running) |
| **Fig. 7-S2L (n=93 BS, 22 NS cells, in 4 mice)** | MU | 5.4 | -21.1 | | 7.2 | -15.1 |  |
|  | SU | -1.0 | -4.2 | | 2.6 | 0.9 |  |

**Figure 7—source data 1.** Results of significance testing across different conditions. Power amplitudes are in units of 1,000*uV^2^/Hz and firing rates are in Hz. MU: multi-unit, SU: single-unit.
